# The role of dog keeping in the home microbiota and its impact on children's health

**DOI:** 10.1111/pai.70408

**Published:** 2026-06-12

**Authors:** Jenni M. Mäki, Pirkka V. Kirjavainen, Martin Täubel, Pauli Tuoresmäki, Eija Piippo‐Savolainen, Katri Backman, Juha Pekkanen, Anne M. Karvonen

**Affiliations:** ^1^ Department of Public Health Finnish Institute for Health and Welfare Kuopio Finland; ^2^ Department of Paediatrics Kuopio University Hospital Kuopio Finland; ^3^ Department of Clinical Nutrition, Institute of Public Health and Clinical Nutrition University of Eastern Finland Kuopio Finland; ^4^ University of Eastern Finland Kuopio Finland; ^5^ Department of Public Health University of Helsinki Helsinki Finland

**Keywords:** children, dog, house dust microbiota, indoor microbiome, respiratory infection

## Abstract

**Background:**

Children with dog contacts in early childhood are healthier and use less antibiotics during the first year of life. We have earlier identified dog‐associated bacterial and fungal signals in house‐dust microbiota. The aim of this study was to determine whether these signals in house dust mediate the dog keeping associated positive health effects in the first year of life.

**Methods:**

We studied 368 Finnish children from pregnancy and collected weekly diary data on otitis, fever, usage of antibiotics, and dog keeping from 9th to 52nd postnatal weeks. The dog‐associated signals in house dust microbiota included the abundance of 12 bacterial and two fungal genera, which were determined alongside richness with Illumina MiSeq sequencing from floor dust samples collected at the child age of 2 months. Bacterial cell concentrations in floor dust were measured with quantitative PCR (qPCR). Generalized estimating equations (GEE) were used for statistical analyses of the microbe‐health associations.

**Results:**

Eight of the dog‐associated genera individually explained 10%–23% of the protective associations between dog keeping and at least one health outcome. The strongest effect was observed for the *Pasteurella* in relation to fever weeks. In addition, three correlated bacterial genera together explained 25% of the dog‐associated reduction in antibiotic use. Bacterial or fungal richness, total cell concentrations, or proportion of human‐sourced bacteria in house dust did not significantly mediate the dog‐associated health effects.

**Conclusions:**

Specific dog‐associated bacterial and fungal genera in house dust, but not richness, explained partly the associations between dog keeping and lower prevalence of respiratory infections.

**Key message**

Dog‐associated microbiota in the house dust affect positively children's health during the first year of life. Dog‐associated bacterial and fungal genera or their combinations in house dust may explain up to 1/5 of the associations between dog keeping and lower prevalence of respiratory infections in the first year of life.

## 1 INTRODUCTION

Exposure to dogs and other pet animals may have positive health effects on human health such as higher frequency of physical activity; children interacting with a dog seem to have increased confidence and prevention of respiratory tract infections^1^, ^2^, ^3^ which are known to increase the risk for childhood asthma.^4^ Some studies suggest that exposure to a dog reduces the risk of allergic diseases,^5^, ^6^, ^7^ while some claim that the dog increases the risk of allergy and asthma in children.^8^ However, it is not known what is behind this phenomenon. One possibility is the microbial exposures in early childhood,^6^, ^9^, ^10^, ^11^ which are known to affect development of the child's immune system and microbiota and therefore the risk of disease.^12^, ^13^ For example, the microbiota in the home has been found to explain some of the protective effects of cattle farms against asthma and allergies.^10^, ^14^

Earlier studies have determined the impact of pets on microbiota of the home^9^, ^11^, ^15^, ^16^, ^17^, ^18^, ^19^, ^20^ and the human gut.^21^, ^22^ Pets alter the microbiota of the homes^9^, ^17^ and exposure to furry pets is seen in the infant gut microbiota composition at 3–4 months of age.^23^ Whether these effects on house dust microbiota mediate the pet‐associated beneficial health effects in children, such as the lower prevalence of respiratory infections, has not been studied.

In the same Finnish birth cohort (LUKAS2), as in the present study,^1^ we previously reported that children who had dog or cat contact(s) at home early in life were mostly healthy, had less fever and otitis, and tended to need fewer courses of antibiotics before the age of 12 months. One plausible mechanism of these pet‐associated beneficial effects is through modifying the early‐life indoor microbial exposures and its consequent beneficial effects on maturation of the immunological defense and human microbiome. We characterized the effects of dog keeping on house dust microbiota in LUKAS2 homes and determined the reproducibility of these effects in a German birth cohort, LISA.^9^ The relative abundances of twelve bacterial and two fungal genera were significantly higher in dog than non‐dog households. In addition, bacterial richness (Chao1) and the concentrations of Gram‐negative bacteria were significantly higher, and the proportion of human‐sourced bacteria as well as the concentration of Gram‐positive bacteria were lower in the households with dog compared to non‐dog households. Fungal richness (Chao1) was higher in the dog homes in LUKAS2, but not in LISA, and the difference was significant only when the ground was covered by snow.

The aim of this study was to determine whether the above‐mentioned bacterial and fungal signatures in house dust could explain the association between dog ownership and the positive health effects in children in the first year of life.

## 2 METHODS

Two birth cohorts (LUKAS1 and LUKAS2) consisting of 442 Finnish children were followed up from the third trimester of pregnancy through to 1 year of age.^24^ All mothers gave birth between September 2002 and May 2005. The ethical permission was granted by the Research Ethics Committee, Hospital District of Northern Savo, Kuopio. A written informed consent was obtained from the parents of the participating children.

Parents filled weekly questionnaires consisting of questions related to infectious symptoms during the past 7 days, children's animal contacts and breastfeeding (exclusive, partial, not breastfed). The diary questionnaires began from the 9th postnatal week and continued up to 1 year of age. Parents were first enquired whether their child had been “hale and hearty” during the last 7 days. If not, parents also answered questions about symptoms and diseases. Based on the earlier report on dog‐associated health effects in this cohort,^1^ the numbers of reported healthy weeks, weeks of antibiotic use, weeks of otitis and weeks of fever were studied (Table 1). The maximum number of diary weeks was 44. We excluded children with less than 23 weeks of diary responses from analyses (*n* = 27) (more information on Data S1 and in Bergroth et al.^1^).

**TABLE 1:** Characteristics of the study population.

	Study population		Diary weeks	
	*N* = 368		*N* = 15,686	
*Characteristic*	*n*	%	*n*	%
Sex				
Male	184	50.0	–	–
Female	184	50.0	–	–
Birth weight				
<3480	120	32.6	–	–
3480–3810	126	32.2	–	–
>3810	122	33.2	–	–
Dog ownership				
No dog	–	–	10,667	68.0
Owning a dog(s)	–	–	5019	32.0
Breastfeeding				
No	–	–	6925	44.2
Partly	–	–	6524	41.6
Solely	–	–	2237	14.3
Number of older siblings				
0	128	34.8	–	–
1	124	33.7	–	–
≥ 2	116	31.5	–	–
Living environment				
Farm	112	30.4	–	–
Rural, non‐farm	176	47.8	–	–
Suburban	80	21.7	–	–
Season of birth				
Winter	101	27.5	–	–
Spring	111	30.2	–	–
Summer	63	17.1	–	–
Autumn	93	25.3	–	–
Maternal smoking^				
No	344	93.5	–	–
Yes	24	6.5	–	–
Parental atopy				
No	168	45.7	–	–
Yes	200	54.4	–	–
Cohort				
LUKAS1	192	52.2	–	–
LUKAS2	176	47.8	–	–
Healthy weeks				
No	–	–	5010	31.9
Yes	–	–	10,676	68.1
Antibiotic usage (weeks)				
No	–	–	15,105	96.3
Yes	–	–	581	3.7
Otitis (weeks)				
No	–	–	14,869	97.4
Yes	–	–	399	2.6
Missing			418	
Fever weeks				
No	–	–	14,631	95.8
Yes	–	–	637	4.2
Missing			418	

*Note*: *N*/*n* the number of observations (in study population) or weeks (in diary weeks) in total and within category; % the percentage of the category. Maternal smoking^ when children were at 2 months old. – indicates missing formation due to the information was collected either in questionnaires (during pregnancy or follow‐up questionnaire at the age of 2 months) or in weekly diary.

### 2.1 Dust sampling

Living room floor dust samples were collected at 2 months of age using a nylon sampling sock and by vacuuming an area of 1 m^2^ from a rug for 2 min or an area of 4 m^2^ from a smooth floor for 2 min. Dust samples were homogenized by sieving through a sterile strainer, dried in a dessicator at 4°C and then stored at −20°C until DNA extraction.

### 2.2 Characterization of the microbes

The house dust microbiota characterization has been described in detail previously.^9^ In brief, DNA from dust samples was extracted using a bead‐beading method, followed by kit‐based clean‐up a concentration. The bacterial/archaeal 16S ribosomal RNA gene V4 region was amplified using 515F/806R primers^25^ and the fungal internal transcribed spacer (ITS) region by ITS1F/ITS2 primers.^26^ The generated DNA amplicons were sequenced with Illumina MiSeq with V3 chemistry. The bioinformatic processing followed the same pipeline as we have earlier described.^9^ In short, the sequences were clustered into operational taxonomic units (OTUs) at 97% similarity using the open‐reference OTU‐picking approach. Richness (Chao1) was calculated with QIIME (Quantitative Insights Into Microbial Ecology).^27^ For the analysis of individual fungal taxa, taxonomic classification was done using FHiTINGS (Fungal High throughput Taxonomy Identification in NGS).^28^ After quality filtering, data on bacterial microbiota was available from the homes of 394 children and on fungal microbiota from the homes of 382 children.

Bacterial concentrations in house dust were measured using quantitative polymerase chain reaction (qPCR) duplex assays for parallel determination of Gram‐positive and Gram‐negative bacterial DNA.^29^ Microbial concentrations were analyzed as cell equivalents per milligram dust (CE/mg). In addition to these, three allergens (cat allergen (Fel d1), dust mite *Dermatophagoides farina* (Der f1), and storage dust mite *Dermatophagoides pteronyssinus* (Der p1)) had been previously determined in LUKAS1 from the 2mo dust sample.

### 2.3 Statistical analyses

The microbial signatures studied in here for the mediation effect were selected based on our earlier findings in LUKAS2.^9^ The selection of microbial genera associated with dog‐households was based on FDR‐corrected *p*‐values in ANCOM analyses.^30^ These included 12 bacterial genus‐level taxa, two fungal genera and bacterial and fungal richness, the concentrations of Gram‐negative and Gram‐positive bacteria, and proportion of human‐sourced bacteria [human source proxy, HSP] in house dust (see more information in the Data S1). Correlations between the different microbial signatures were calculated using Spearman's rank correlation, which were also used for network analyses for studying taxa interactions (*ρ* ≥ 0.5). For statistical analyses, the microbial signatures were divided into tertiles (see descriptives in Table S1), except HSP, for which the ln‐logarithm variable was used, and the results were expressed per interquartile range (IQR) change. If the percentage of samples under detection level was above 33.3%, then the values above detection level were divided into two equal classes using median as cut off. Generalized estimating equations (GEE) method with working correlation matrix AR (1) was used for statistical analyses. In the mediation analyses, the percentage change in the dog's estimate was calculated from estimates given by two models: (1) an original model in which the confounding factors were adjusted and (2) the original model in which was additionally adjusted for a microbial signature. All models were adjusted similarly as in Bergroth et al.^1^ in order to study the mediation effect of the dog's microbiota on the previously reported inverse association between the dog and respiratory infections. A priori selected adjustments were related to general risk factors for allergic diseases gender, number of older siblings (none, 1, ≥2), maternal smoking (children age 2 months), parental atopy, weekly data on breastfeeding (exclusive, partial or not breastfed), birth weight (in tertiles), and season of birth (winter, spring, summer, autumn) and study characteristics (living environment (farm, rural non‐farm, suburban), the order of diary month, and cohort (LUKAS1, LUKAS2)).

## 3 RESULTS

A total of 15,686 diary weeks were recorded from the 368 children who had information on any of the four outcomes and on dog keeping and either bacterial or fungal sequencing data and had no missing data on a priori selected confounders (Table 1). The study sample comprised an equal number of boys and girls. Twenty‐four mothers (6.5%) smoked, and two of them smoked indoors. Of the children, 112 (30.4%) lived on a farm, 176 (47.8%) in a rural area (non‐farm), and 80 (21.7%) in a suburban area.

Of the 14 dog‐associated genera, *Clostridium* and *Fusobacterium* had the highest median abundance in dust samples (Table S1). All of the 12 bacterial genera correlated positively with each other (mostly *ρ* = 0.2–0.4); correlations with the two fungal genera were weaker, though still largely positive (Figure S1). We observed the strongest correlations (*ρ* >0.7) between *Pasteurella*, *Lampropedia*, and *Conchiformibius* genera.

### 3.1 Associations between 14 genera and four health outcomes

**FIGURE 1:**
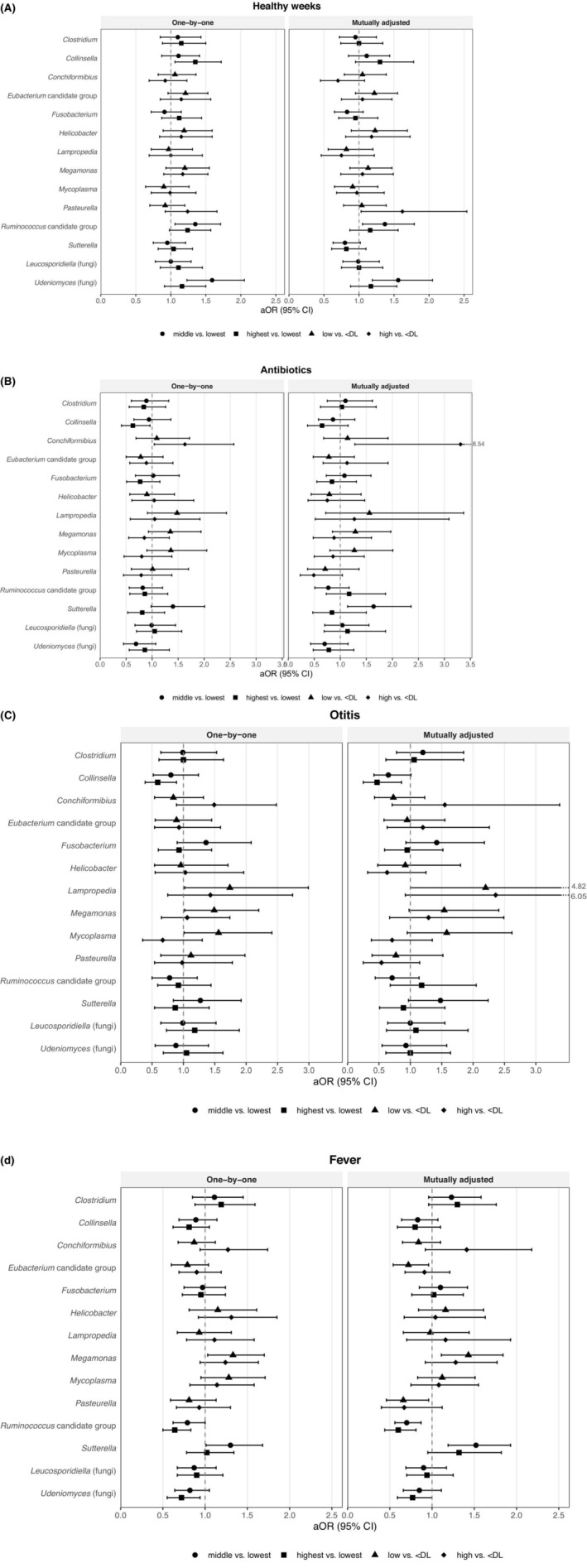
Individually (one‐by‐one) and jointly (mutually) adjusted models of associations between 14 dog‐associated bacterial and fungal genera in house dust and (A) healthy weeks, (B) use of antibiotics, (C) otitis, and (D) fever weeks in children aged 2–12 months. AOR Adjusted Odds Ratios; 95% CI Confidence intervals obtained by using GEE analysis, working correlation matrix AR (1). Genera are divided into tertiles/categorized into three classes (under detection level, and two equal classes using median as cut off) and the lowest category is the reference category. Models are adjusted for dog ownership (weekly), gender, birth weight, number of older siblings, living environment, season of birth, diary month, breastfeeding (weekly), maternal smoking and parental atopy and cohort. Total number of diary weeks in bacterial analyses in healthy weeks and usage of antibiotics is 15,644 and in otitis and fever weeks is 15,226 and in fungal analyses the similar values are 15,129 and 14,720, which correspond to the numbers of 367 and 355 children, respectively.

The higher levels of *Collinsella* were positively associated with healthy weeks (Figure 1A) and inversely with the use of antibiotics (Figure 1B) and weeks with otitis (Figure 1C) when dog keeping was taken account (Trend test *p < .05*, Table S2), and a similar tendency (*p = .11*) was seen with fever weeks (Figure 1D). The higher levels of *Ruminococcus* candidate group and *Udeniomyces* were inversely associated with fever weeks. There was a tendency that higher levels of *Conchiformibius* and of *Megamonas* were associated with more antibiotic treatment weeks and more fever weeks (*p = .09* and *0.07*), respectively.

In models including all 14 genera simultaneously, *Collinsella* was, independently of the other genera, positively associated with healthy weeks (Figure 1A) and inversely with weeks with otitis (Figure 1C); *Pasteurella* was inversely associated with the antibiotic treatment weeks (Figure 1B) and weeks with fever (Figure 1D), and *Ruminococcus* candidate group was inversely associated with fever weeks (*p < .05*, Table S3).

### 3.2 How much 14 different genera explain the association between a dog and a health effect?

**FIGURE 2:**
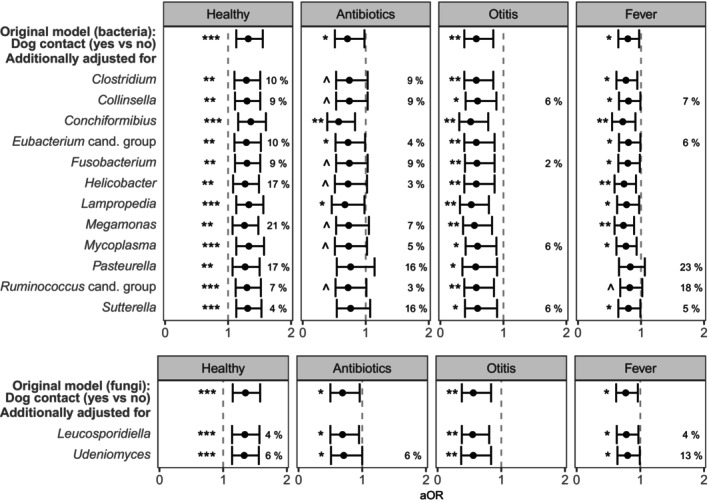
Adjusted associations between dog ownership and healthy weeks, usage of antibiotics, weeks of otitis or fever, additionally adjusted for the bacterial and fungal genera measured in house dust. AOR adjusted Odds Ratios, whiskers indicate the 95% confidence intervals obtained by using GEE analysis, working correlation matrix AR (1), and group means candidate group; original models are adjusted for gender, birth weight, number of older siblings, living environment, season of birth, diary month, breastfeeding (weekly), maternal smoking, and parental atopy and cohort and additionally with the given genus (categorized into three classes). % percentage of the genus explains the association between dog contact and the given outcome. If % is missing, the genus does not explain the association. Total number of diary weeks in bacterial analyses in healthy weeks and usage of antibiotics is 15,644 and in otitis and fever weeks is 15,226, and in fungal analyses the similar values are 15,129 and 14,720, which correspond to the numbers of 367 and 355 children, respectively. ^ *p*‐value <0.1, * < 0.05, ** < 0.01, *** < 0.001.

Out of the 14 genera, *Conchiformibius* and *Lampropedia* did not explain the dog effect, whereas *Collinsella*, *Fusobacterium*, *Mycoplasma*, and *Leucosporidiella* explained only a minor part (<10%), and the rest of the eight genera each explained 10%–23% of the association between dog keeping and at least one of the studied outcomes (Figure 2), which were in healthy weeks: *Clostridium, Eubacterium* candidate group, *Helicobacter*, *Megamonas*, and *Pasteurella*; in antibiotic treatment weeks: *Pasteurella* and *Sutterella*; and in fever weeks: *Pasteurella, Ruminococcus* candidate group, and *Udeniomyces*. None of the genera explained the association between dog and otitis more than 6%. In addition, the inverse associations between dog ownership and the use of antibiotics were no longer significant after adjustment with *Pasteurella* or *Sutterella*, nor were fever weeks after adjustment with *Pasteurella* (*p > .1*, Figure 2).

The simultaneous adjustment for the 14 genera did not, per se, explain the dog‐associated associations with the health outcomes, but rather fortified these associations, except for healthy weeks, for which the effect remained unchanged (Table S3).

Due to the co‐occurrence of the genera, we checked whether dog‐associated genera together could explain more of the health benefits of dog keeping than the single genera, and thus, we used two methods: (1) correlation‐based network analyses and (2) summing up the selected genera. The first method distinguished two groups of three bacterial genera (Figure S2). When the first group (A), which included *Collinsella, Ruminococcus* candidate group, and *Sutterella*, was adjusted in the model simultaneously with their interaction terms, they explained 2%–25% of the inverse association between the dog and respiratory infections (Table 2). However, the second group (B) (*Lampropedia, Pasteurella*, and *Conchiformibius*) did not explain the association but rather strengthened the protective effect of the dog. In the second method, the relative abundances of 2–7 genera, which were significantly (Trend test *p < .1*) associated with a health outcome when dog keeping was not taken into account, were summed up (Table S4). The sums of selected genera were for ‘healthy weeks’ *Clostridium, Collinsella, Eubacterium* candidate group, *Helicobacter, Megamonas*, *Pasteurella*, and *Ruminococcus* candidate group; for the ‘antibiotic treatment weeks’ *Collinsella* and *Pasteurella*; and for the ‘weeks of fever’ *Collinsella, Pasteurella, Ruminococcus* candidate group, and *Udeniomyces*. Only *Collinsella* genus was significantly associated with otitis and thus, the sum variable was not created. The sum variables were significantly associated with given outcomes (Table S4) and similar tendency (*p* ≤ .10) were seen when dog keeping was taken account (Table S5). The sum variables did not explain more the effect between dog keeping and health benefits than the single genera did: the sum of selected genera for healthy, for antibiotic treatment, and for fever weeks explained 21%, 17%, and 15%, respectively.

**TABLE 2:** Effect of two groups of three correlated bacterial genera—identified via correlation‐based network analyses (Spearman correlation ≥0.5)—on the dog‐associated reduction in respiratory infections.

Original model: dog contact (yes vs. no)	Healthy				Antibiotics				Otitis				Fever			
	aOR	(95% CI)	*p*	Group explains (%)	aOR	(95% CI)	*p*	Group explains (%)	aOR	(95% CI)	*p*	Group explains (%)	aOR	(95% CI)	*p*	Group explains (%)
	1.32	(1.13–1.55)	***		0.71	(0.51–0.98)	*		0.57	(0.38–0.84)	**		0.79	(0.64–0.97)	*	
Group A	1.31	(1.12, 1.52)	***	5%	0.76	(0.54, 1.06)		16%	0.60	(0.40, 0.90)	*	6%	0.81	(0.65, 1.00)	*	11%
Group A + their 3 interaction terms	1.31	(1.12, 1.53)	***	5%	0.78	(0.56, 1.10)		25%	0.62	(0.42, 0.91)	*	12%	0.79	(0.64, 0.97)	*	2%
Group B	1.35	(1.14, 1.60)	***		0.61	(0.41, 0.90)	*		0.49	(0.30, 0.80)	**		0.74	(0.57, 0.96)	*	
Group B + their 3 interaction terms	1.35	(1.15, 1.60)	***		–				–				–			

*Note*: Group A: *Collinsella, Ruminococcus* candidate group and *Sutterella*; Group B: *Lampropedia, Pasteurella* and *Conchiformibius*; In the first models, the three bacterial genera from either group A or B were adjusted simultaneously in the model together with sex, living environment (farm, rural non‐farm, suburban), number of siblings, maternal smoking, parental atopy, breastfeeding (solely, partly, no), birth weight, season of birth, diary month and cohort. In the second models, the interaction terms of the three bacterial genera were included into the model. % percentage of the three genera jointly explains the association between dog contact and the given outcome. If % is missing, the given group of three genera does not explain the association. *p*‐value: * < 0.05, ** < 0.01, *** < 0.001.

### 3.3 Other microbial signatures and four health outcomes

Among the bacterial loads and richness in house dust, only the concentration of Gram‐negative bacteria (measured via qPCR) was directly associated with increased risk of antibiotic use (Trend test *p = .05*, Table S6). Fungal richness was directly associated with weeks of otitis, but with no other health outcomes (Table S7). None of these microbial measurements explained a considerable portion of the association between dog keeping and health outcomes (Tables S6 and S7).

### 3.4 Sensitivity analyses

In addition to microbial pathway, we were able to study psychological (maternal self‐reported depression and stress) and allergen exposure pathways in either cohort. Maternal self‐reported depression and stress, and measured levels of cat allergen and two dust mites from the dust sample did not differ significantly between households with or without dog (Table S8). However, the storage dust mite (*Dermatophagoides pteronyssinus*) tended to be more often detected in households with dog than in households without dog (9.2% vs. 3.2% *p* = .09, respectively). Neither of them mediated the dog effect and weeks of fever more than 9%. Finally, we conducted analyses using two different imputation methods for the absence of microbial data: a separate class for missing data (Table S9) and the addition of a randomized value for missing observations (Table S10). In the first method, the results were similar to those in original models. In the latter, *Clostridium*, *Eubacterium* candidate group and *Pasteurella* in healthy weeks, and *Pasteurella* and *Sutterella* in usage of antibiotics explained the effect less than in original model (around 5%).

## 4 DISCUSSION

We show in this study that dog‐associated bacterial and fungal genera or their combinations in house dust can explain up to 1/4 of the associations between dog keeping and lower prevalence of respiratory infection during the first year of life. We observed the greatest health‐promoting effect with the *Pasteurella* genus with fever weeks. Even though dog keeping increased the levels of microbial richness or total loads measured from house dust, they did not explain the effect.

Perinatal exposure to indoor pets, mainly dogs and cats, was found to be a protective factor against wheezy bronchitis under 2 years of age in another Finnish birth cohort study.^31^ Similarly, we have shown that under one‐year‐old children with dog(s) or cat(s) at home were significantly healthier and had fewer weeks with fever and otitis, and they tended to need less antibiotics during the first year of life.^1^ Due to the stronger protective effect of dogs than cats, we previously studied and identified bacterial and fungal characteristics associated with dog ownership within half of the current study population and replicated the results in a German birth cohort.^9^ In the current study, we found that eight tested dog‐associated genera mediated partially the dog effect, explaining 10–23% of the association between dog keeping and at least one of the studied outcomes. Only a few of the genera had positive effects on health, independently of dog keeping, *Collinsella* being the most consistent in all four outcomes. It seems that certain dog‐associated microbes support children's health and provide strong protection against upper respiratory tract infections at a time when the development of immunological response is at its most sensitive. Although the percentage of mediation that was observed was rather small, this is the first indication that microbial exposure seems to play a role in the protective effect of dog keeping. In addition, we acknowledge that we only study the proxy of true microbial exposure; due to in direct contact with a dog, the mediation effect can be much more significant. Further studies should determine whether dog‐associated microbiota and its health‐protective effect persist beyond the first year of age, whether dog‐associated microbiota can be detected in the respiratory tract of infants, and whether the mediation effect can be confirmed in other cohorts.

*Pasteurella* species are gram‐negative coccobacilli,^32^ that colonize the oral cavity of dogs and cats.^32^, ^33^ They are occasionally found in dogs' inner ear and chin^34^ but typically not present in the normal microbial flora of humans.^35^ Gut microbiota overlap between dogs and their owners has been attributed to shared living environments, partially outdoor‐oriented lifestyles, and convergent dietary patterns.^21^, ^23^, ^31^, ^36^ *Ruminococcus* ^21^ and *Collinsella* ^37^ in the gut are linked to lower childhood obesity risk, with *Ruminococcus* also protective against atopy.^21^ In adults, higher fecal levels of these genera correlated with reduced severity of COVID‐19.^38^, ^39^ However, the potential impact of these genera, including dog‐associated taxa such as *Megamonas, Helicobacter, Pasteurella*, and *Udeniomyces*, on early‐life respiratory infections is still unknown.

Prior research indicates that early exposure to dogs is associated with a reduced risk of developing asthma,^6^ allergicy,^6^ atopic dermatitis,^40^ childhood lymphoblastic leukemia,^41^ as well as neuropsychiatric disorders such as schizophrenia^42^ and bipolar disorder.^42^ Evidence on the impact of pet dogs on infant respiratory microbiota is limited,^15^ however, farm exposure has been studied in older children and adults.^43^, ^44^, ^45^ How much environmental microbes, especially associated to pet dogs, determine the composition of the respiratory tract at early age and how that microbiota changes after birth, is a less studied area compared to studies of the intestinal microbiota.^46^ Earlier studies have suggested that allergies and childhood leukemia might share a common etiological basis, implying that early‐life immune dysregulation could play a role in the pathogenesis of both conditions.^47^ Exposure to a pet dog during fetal development and early childhood has been hypothesized to influence gut microbiota composition,^42^ which in turn may confer protection against schizophrenia through changes in the brain‐immune‐gut axis.^48^ Our study extends previous research by demonstrating that certain dog‐associated microbes provide protection against early childhood infections, thereby reducing the risk of asthma and allergies, and potentially offering a pathway through which the risk of neuropsychiatric disorders could also be mitigated.

We observed similar health‐promoting associations with a single genus than with a combination of selected bacterial and fungal genera, but no similar protective or mediating effect was seen with overall bacterial and fungal richness or total amounts of Gram‐positive or Gram‐negative bacteria (measured via qPCRs) in house dust. As far as we know, no other study has investigated the association between dog keeping and house dust microbiota and its association with human respiratory infections. Although in our study the dog increased the levels of richness or load of bacteria in house dust or decreased the human‐associated bacteria, they did not explain the protective effect of the dog on respiratory infections.

In this study, the bacterial and fungal genera in house dust explained the protective effect of the dog on otitis less than on other health outcomes. Acute otitis is usually a viral‐bacterial co‐infection.^49^ Parents reported otitis diagnosed by a doctor and the otitis diagnosis was not verified from health care records. Otitis can heal on its own without antibiotics, and a child with mild symptoms can have otitis without knowing it. Therefore, it is possible that not all cases of otitis have been reported. Mild cases could have been missed, symptoms forgotten, or non‐specific signs such as fever and irritability misattributed, leading to potential misclassification. However, weekly reporting likely improves reliability compared with longer recall periods, and any reporting bias should be similar across exposure groups, minimizing its impact on the results.

The strength of our study was sequenced house dust samples from a relatively large study population with approximately one‐third of dog homes. Another strength of our research was a weekly questionnaire about the child's health filled in by the parents during the first year of age. The first year of age is an important time window in terms of the development of the child's immunology. By combining this information, we were able to study the effect of certain dog‐associated microbes on the health of children under 1 year of age. The weaknesses of our study were related to samples: only one dust sample was collected, no information on viruses was available, nor were samples collected from children's airways. Although seasonal variation cannot be entirely controlled for with a single sample even though the season was adjusted in the models, dust from floors/rugs at 2 months can still provide a reasonably representative index of long‐term microbial exposure during early life. We acknowledge that our analysis is limited to taxonomic profiling of microbial communities in house dust, which allows little functional prediction given that a large proportion of DNA signal will not be associated with metabolically active microbes.^50^ To advance this field we recommend that future studies will consider metabolite profiling and/or metatranscriptomics to validate functional mechanisms underlying the observed health associations. Also, our findings represent one environmental and cultural context; however, replication in other populations in different contexts would have strengthened external validity. We also acknowledge that the development of the immune system is the sum of many factors, which cannot all be taken into account, though we adjusted models with several confounding factors. Observational study also cannot show cause‐effect relationship. There are other positive factors associated with owning a dog than the abundance of home indoor microbiota shaped by a dog, for example an outdoor‐oriented lifestyle, structured schedules (e.g. walking times and feeding), and dog ownership‐related psychological factors (shared responsibilities), which can improve family bonds. Through their influence on direct and indirect microbial exposures and human behavior, dogs may mitigate the negative health effects of an urbanized environment. However, the results of the mediation analyses are informative regarding potential pathways and should be viewed with the usual caution applied to non‐experimental data.

In conclusion, specific dog‐associated bacterial and fungal genera in house dust, but not richness, explained partly the associations between dog keeping and lower prevalence of respiratory infections. However, the replicability of the mediation effect remains to be confirmed in other cohorts.

## AUTHOR CONTRIBUTIONS

**Pirkka V. Kirjavainen:** Conceptualization; writing – original draft; writing – review and editing; methodology. **Anne M. Karvonen:** Conceptualization; funding acquisition; writing – original draft; writing – review and editing; visualization; methodology; project administration; supervision. **Eija Piippo‐Savolainen:** Writing – review and editing. **Juha Pekkanen:** Conceptualization; writing – original draft; writing – review and editing; project administration; supervision; methodology; funding acquisition. **Katri Backman:** Writing – review and editing. **Pauli Tuoresmäki:** Visualization; software. **Martin Täubel:** Conceptualization; writing – original draft; writing – review and editing; methodology. **Jenni M. Mäki:** Conceptualization; writing – original draft; writing – review and editing; methodology; visualization.

## FUNDING INFORMATION

The microbiota sequencing and analyses were supported by Academy of Finland as part of PROBIOM consortium Project (296814, 296817), and Juha Vainio Säätiö (201710468). The LUKAS work was supported by the research Grants from the Academy of Finland (Grants 139021; 287675; 308253; 308254); the Juho Vainio Foundation; Päivikki and Sakari Sohlberg Foundation; the Foundation for Pediatric Research; EVO/VTR‐funding; the Yrjö Jahnsson Foundation; Maatalousyrittäjien eläkelaitos ‐ Mela; The Finnish Cultural Foundation; European Union (QLK4‐2001‐00250 and FP7‐211911); Kuopio Area Respiratory Foundation; Tampere Tuberculosis Foundation; the Finnish Institute for Health and Welfare, Finland. The funding organizations were not involved in the design of the study and the collection, analysis, and interpretation of data or in writing the manuscript.

## CONFLICT OF INTEREST STATEMENT

The authors have no conflicts of interest to disclose.

## Supporting information


**Table S1:** Description of the levels of bacterial and fungal parameters in dust samples from living room floors collected at the age of 2 months in LUKAS1 and LUKAS2.
**Table S2:** Adjusted associations between relative abundances of dog‐associated 12 bacterial or two fungal genera in dust samples and healthy weeks, usage of antibiotics, weeks of otitis or fever.
**Table S3:** Adjusted associations between dog keeping and healthy weeks, usage of antibiotics, weeks of otitis or fever, and simultaneously adjusted 14 relative abundances of bacterial and fungal genera in dust from living room floor.
**Table S4:** Adjusted associations between relative abundances of dog‐associated 12 bacterial or 2 fungal genera as well as sum of selected genera in dust samples and healthy weeks, usage of antibiotics, weeks of otitis or fever in children aged 2–12 months of age.
**Table S5:** Adjusted associations between dog keeping and healthy weeks, usage of antibiotics, or fever weeks, and mutually adjusted for sum of selected bacterial and fungal genera in dust from living room floor.
**Table S6:** Adjusted associations between dog keeping and healthy weeks, usage of antibiotics, weeks of otitis or fever, additionally adjusted for bacterial signatures in house dust.
**Table S7:** Adjusted associations between dog keeping and healthy weeks, usage of antibiotics, weeks of otitis or fever, additionally adjusted for fungal richness in house dust.
**Table S8:** Additional characteristics and comparisons between households with and without dogs.
**Table S9:** Adjusted associations between dog keeping and healthy weeks, usage of antibiotics, weeks of otitis or fever, additionally adjusted for bacterial and fungal genera in dust from living room floor (separate class for missing added).
**Table S10:** Adjusted associations between dog keeping and healthy weeks, usage of antibiotics, weeks of otitis or fever, additionally adjusted for bacterial and fungal genera in dust from living room floor (missing input at random).
**Figure S1:** Heat map of Spearman correlations between bacterial and fungal signatures or genera in LUKAS cohorts (LUKAS1 + 2) of 394 dust samples. Heat map was created using R software. (%) defines relative abundance; qPCR quantitative polymerase chain reaction; CE cell equivalent; mg per milligram of dust. The sum of relative abundances of genera was *Clostridium, Collinsella, Eubacterium* candidate group, *Helicobacter, Megamonas, Pasteurella*, and *Rumninococcus* candidate group in healthy weeks; *Collinsella* and *Pasteurella* in usage of antibiotics; and *Collinsella, Pasteurella, Rumninococcus* candidate group and *Udeniomyces* in weeks of fever. No sum variable for otits was created.
**Figure S2:** Result from the correlation‐based network analyses. Group A consist of *Collinsella, Ruminococcus* candidate group (*) and *Sutterella* and the group B *Lampropedia, Pasteurella* and *Conchiformibius*.

## Data Availability

The data that support the findings of this study are available from the corresponding author upon reasonable request.
