# Supplementary material for: The role of dog keeping in the home microbiota and its impact on children's health
Source: Pediatr Allergy Immunol. 2026 Jun 12;37(6):e70408. doi: 10.1111/pai.70408 (PMC13263413; doi:10.1111/pai.70408)
Supplement: Supplementary file 1 — Table S1: Description of the levels of bacterial and fungal parameters in dust samples from living room floors collected at the age of 2 months in LUKAS1 and LUKAS2. Table S2: Adjusted associations between relative abundances of dog‐associated 12 bacterial or two fungal genera in dust samples and healthy weeks, usage of antibiotics, weeks of otitis or fever. Table S3: Adjusted associations between dog keeping and healthy weeks, usage of antibiotics, weeks of otitis or fever, and simultaneously adjusted 14 relative abundances of bacterial and fungal genera in dust from living room floor. Table S4: Adjusted associations between relative abundances of dog‐associated 12 bacterial or 2 fungal genera as well as sum of selected genera in dust samples and healthy weeks, usage of antibiotics, weeks of otitis or fever in children aged 2–12 months of age. Table S5: Adjusted associations between dog keeping and healthy weeks, usage of antibiotics, or fever weeks, and mutually adjusted for sum of selected bacterial and fungal genera in dust from living room floor. Table S6: Adjusted associations between dog keeping and healthy weeks, usage of antibiotics, weeks of otitis or fever, additionally adjusted for bacterial signatures in house dust. Table S7: Adjusted associations between dog keeping and healthy weeks, usage of antibiotics, weeks of otitis or fever, additionally adjusted for fungal richness in house dust. Table S8: Additional characteristics and comparisons between households with and without dogs. Table S9: Adjusted associations between dog keeping and healthy weeks, usage of antibiotics, weeks of otitis or fever, additionally adjusted for bacterial and fungal genera in dust from living room floor (separate class for missing added). Table S10: Adjusted associations between dog keeping and healthy weeks, usage of antibiotics, weeks of otitis or fever, additionally adjusted for bacterial and fungal genera in dust from living room floor (missing input at random). Figure S [file PAI-37-e70408-s001.docx]

SUPPLEMENTARY MATERIAL

**The role of dog keeping in the home microbiota and its impact on children’s health**

Jenni M. Mäki, Pirkka V. Kirjavainen, Martin Täubel, Pauli Tuoresmäki, Eija Piippo-Savolainen, Katri Backman, Juha Pekkanen, Anne M. Karvonen

Contents

[Methods 2](#_Toc225791498)

[Tables and figures 6](#_Toc225791499)

[Supplemental Table E1 6](#_Toc225791500)

[Supplemental Table E2. 7](#_Toc225791501)

[Supplemental Table E3 9](#_Toc225791502)

[Supplemental Table E4 11](#_Toc225791503)

[Supplemental Table E5. 13](#_Toc225791504)

[Supplemental Table E6 14](#_Toc225791505)

[Supplemental Table E7 15](#_Toc225791506)

[Supplemental Table E8 16](#_Toc225791507)

[Supplemental Table E9 17](#_Toc225791508)

[Supplemental Table E10 18](#_Toc225791509)

[Supplemental Figure E1. 19](#_Toc225791510)

[Supplemental Figure E2 21](#_Toc225791511)

# Methods

Two birth cohorts (LUKAS1 and LUKAS2) consisting of 442 Finnish children were followed up from the third trimester of pregnancy through to one year of age.^1^ All mothers gave birth between September 2002 and May 2005. The ethical permission was granted by Ma (IORG00005196, LUKAS1:299/2017(33/2002), LUKAS2:300/2017(48/2004)). A written informed consent was obtained from parents of the participating children.

***Weekly diary***

Parents filled weekly questionnaires consisting of questions related to infectious symptoms during the past seven days, children’s animal contacts and breastfeeding (exclusive, partial, not breastfed). The diary questionnaires began from the 9^th^ postnatal week and continued up to one year of age. Based on the earlier report on dog-associated health effects in this cohort,^2^ parents were first enquired whether their child had been “hale and hearty” during the last 7 days. If not, parents also answered questions about symptoms and diseases (whether the child had had cough, wheezing of breath, rhinitis, fever 38.5°C, middle ear infection, diarrhea, urinary tract infection, itchy rash, or some other illness during these last 7 days). We also asked whether there was a dog(s) or a cat(s) inside the home. Based on the earlier report on dog-associated health effects in this cohort,^3^ the numbers of reported healthy weeks, weeks of antibiotic use, weeks of otitis and weeks of fever were studied. The maximum number of diary weeks were 44. We excluded children with less than 23 weeks of diary responses from analyses (n=27). When data on animal contacts or information on the health, respiratory symptoms, and infections were missing from a diary, the week in question was excluded from the analysis. The same was done with other missing data if the information was not reliably derivable from other sources (eg, missing breastfeeding status from previous or subsequent weeks). Based on the earlier report, ^3^ information was originally received for a total of 17 124 follow-up weeks recorded from the 397 children. More weekly questionnaires were completed at the beginning of the follow-up: the number varied from 396 forms in weeks 7, 11, 12, 17, and 18 to 347 forms in week 44. Of the 44 follow-up weeks, the mean number of completed weeks was 43.0 per child (range: 23–44 weeks), with 94.2% of families filling in at least 40 forms. In the current paper, depending on the health outcomes and either bacterial or fungal signatures or both, the total number of diary weeks varied between 15 644 and 14 678, corresponding to 367 and 354 children, respectively.

***Season in dust sampling***

Seasons of dust sampling were based on the date of house dust sampling and categorized as follows: winter between December-February, spring March-May, summer June-August and autumn September-November. When LUKAS2 children were born, the study area was covered by snow from December 15^th^2004 until the end of March 2005 (Finnish National Climatology. https://www.ilmatieteenlaitos.fi/talvi-2004-2005). Since flooring and in particular rugs are dust reservoirs and samples represent dust accumulated over time, we decided to use two weeks lag-period after snow started covering the ground before categorizing the sampling done during snow cover (from January 1^st^2004 to 31^st^ March 2005). This information was used for stratified analyses by snow cover in the Mäki et al. 2021.

***Human source proxy (HSP)***

An index reflecting the relative abundance of bacteria likely originating from human microbiota (human source proxy, HSP) was in our previous study^3^ created by summing up the relative abundances of two human microbiota associated genera (*Staphylococcus* and *Streptococcus*) and three families (*Corynebacteriaceae, Propionibacteriaceae*, and *Enterobacteriaceae*).

***Dog associated microbial signatures based in our previous study***

In our previous publication,^3^ bacterial richness (Chao1) and the concentrations of Gram-negative bacteria in house dust (cell equivalent per mg of dust) were significantly higher in the dog homes compared to non-dog homes. The relative abundance of human-associated bacteria (HSP) and the concentration of Gram-positive bacteria were lower in the dog than non-dog homes. In analysis stratified by snow cover bacterial richness was significantly higher in the dog homes only when the ground was not covered by snow.^3^ The higher concentrations of Gram-negative bacteria in the dog than non-dog homes were more pronounced during snow cover and the lower level of Gram-positive bacteria during no snow cover. Fungal richness (Chao1) was higher in the dog homes in LUKAS2, but not in LISA and the difference was significant only when the ground was covered by snow.

In the same publication,^3^ we identified 12 dog-associated bacterial and two fungal genera, which relative abundance was higher in homes with dog(s) than homes without dog(s): *Clostridium*, *Conchiformibius, Helicobacter, Megamonas*, *Mycoplasma, Pasteurella* and *Leucosporidiella.* When snow covered the ground, genera *Collinsella*, *Eubacterium* candidate group, *Fusobacterium,* *Ruminococcus* candidate group, *Sutterella* and *Udeniomyces* were more abundant in dog homes. Of those genera, six assigned genera were replicated (i.e. were more abundant in dog homes) in a German LISA birth cohort with dust sample collected at the age of 3 months. These genera were within phylum Firmicutes: *Clostridium* and *Megamonas*, genera within p. Proteobacteria: *Conchiformibius, Helicobacter, Pasteurella*; and *Mycoplasma* (p. Tenericutes).

***Other possible pathways —*** ***psychological, allergen exposure — alongside microbial factors***

With this LUKAS1 and LUKAS2 data, we were able to study two additional pathways alongside microbial factors: psychological (maternal self-reported depression and stress) and allergen exposure. Maternal self-reported depression and stress at 1-year questionnaire was enquired only in LUKAS2, and allergen levels were measured from the 2mo house dust sample only in LUKAS1: cat allergen (Fel d 1), and the mount of two dust mites from the same dust sample (*Dermatophagoides farina* (Der f) and *Dermatophagoides pteronyssinus* (Der p)). Maternal mental well-being and stress were asked using questionnaires when the child was 1-year old with followed questions: “Have you recently felt persistently overwhelmed?” and “Have you recently felt unhappy and depressed?” and the answer options were in both questions: Not at all/ not more than usual / more than usual / much more than usual. If either of the answers were more than usual or much more than usual, the answer was coded as yes.

***Statistical analyses***

The microbial signatures were selected based on our earlier findings in LUKAS2.^2^ These included 12 bacterial genus level taxa (*Clostridium*, *Collinsella, Conchiformibius, Eubacterium* candidate group, *Fusobacterium*, *Helicobacter, Megamonas*, *Mycoplasma*, *Lampropedia, Pasteurella, Ruminococcus* candidate group and *Sutterella*), two fungal genera (*Leucosporidiella* and *Udeniomyces)* and bacterial and fungal α-diversity, the concentrations of Gram-negative and positive bacteria, and HSP in house dust). Correlations between the different microbial signatures were calculated using Spearman’s rank correlation. For statistical analyses the microbial signatures were divided into tertiles, except HSP, which the ln-logarithm variable was used, and the results were expressed as per interquartile range (IQR) change. If the percentage of samples under detection level were above 33.3%, then the values above detection level were divided into two equal classes using median as cut off. Generalized estimating equations (GEE) method with working correlation matrix AR(1) was used for statistical analyses. In the mediation analyses, the percentage change in the dog's estimate was calculated from estimates given by two models: 1) an original model in which the confounding factors were adjusted and 2) the original model in which was additionally adjusted for a microbial signature. All models were adjusted as in Bergroth et al. (2012): gender, living environment (farm, rural non-farm, suburban), number of older siblings (none, 1, ≥2), maternal smoking (children age 2 months), parental atopy, weekly information on breastfeeding (exclusive, partial or not breastfed), birth weight (in tertiles), season of birth (winter, spring, summer, autumn), the order of diary month and cohort (LUKAS1, LUKAS2). Some of the confounding factors were asked from mothers using questionnaires during pregnancy and when the children were 2 months old. In the final models, depending on the health outcomes and either bacterial or fungal signatures or both, the total number of diary weeks varied between 15 644 and 14 678, corresponding to 367 and 354 children, respectively.

**References**

1. Karvonen AM, Hyvärinen A, Roponen M, et al. Confirmed moisture damage at home, respiratory symptoms and atopy in early life: a birth-cohort study. *Pediatrics*. 2009;124(2):e329-338. doi:10.1542/peds.2008-1590

2. Bergroth E, Remes S, Pekkanen J, Kauppila T, Büchele G, Keski-Nisula L. Respiratory tract illnesses during the first year of life: effect of dog and cat contacts. *Pediatrics*. 2012;130(2):211-220. doi:10.1542/peds.2011-2825

3. Mäki JM, Kirjavainen PV, Täubel M, et al. Associations between dog keeping and indoor dust microbiota. *Scientific reports*. 2021;11(1):5341. doi:10.1038/s41598-021-84790-w

# Tables and figures

Supplemental Table E1. Description of the levels of bacterial and fungal parameters in dust samples from living room floors collected at the age of 2 months in LUKAS1 and LUKAS2.

Std Dev = Standard deviation, <DL = under detection limit, freq.= frequency. Genera, human-sourced bacteria, and sum of genera variables are expressed as relative abundances (%); qPCRs as cell equivalents per milligram dust (CE/mg). The genera which were summed up were *Ruminococcus* candidate group and *Udeniomyces* in healthy weeks; *Collinsella* and *Conchiformibius* in usage of antibiotics; *Collinsella,* *Megamonas* and *Mycoplasma* in weeks of otitis; and *Conchiformibius, Eubacterium* candidate group, *Megamonas, Pasteurella*, *Ruminococcus* candidate group, *Sutterella* and *Udeniomyces* in weeks of fever

Supplemental Table E2. Adjusted associations between relative abundances of dog-associated 12 bacterial or two fungal genera in dust samples and healthy weeks, usage of antibiotics, weeks of otitis or fever.

N total number of observations in the analyses; n total number of outcome weeks in the given microbial class; % percentage of the outcome in the given microbial class; aOR Adjusted Odds Ratios; 95% CI Confidence intervals obtained by using GEE analysis, working correlation matrix AR(1). p values are from Trend test; p-values <0.05 are in bold. Genera were divided into tertiles/ categorized into three classes (under detection level, and two equal classes) and the lowest category is the reference category; DL detection limit; Models are adjusted for gender, birth weight, number of siblings, living environment, season of birth, diary month, maternal smoking and parental atopy, cohort and dog ownership. Total number of diary weeks in bacterial analyses in healthy weeks and usage of antibiotics is 15 644 and in otitis and fever weeks is 15 226 and in fungal analyses the similar values are 15 129 and 14 720. The total number of children with diary data was 367 in bacterial and 355 in fungal analyses.

Supplemental Table E3. Adjusted associations between dog keeping and healthy weeks, usage of antibiotics, weeks of otitis or fever, and simultaneously adjusted 14 relative abundances of bacterial and fungal genera in dust from living room floor.

aOR adjusted Odds Ratios; 95% CI, Confidence intervals were obtained by using GEE analysis, working correlation matrix AR(1); p-values for microbial genera are from linear trend test; p-values <0.05 are in bold. DL detection limit; Genera were divided into tertiles/ categorized into three classes (under detection level, and two equal classes) and the lowest category is the reference category; Model 1 was adjusted for sex, living environment (farm, rural non-farm, suburban), number of siblings, maternal smoking, parental atopy, breastfeeding (solely, partly, no), birth weight, season of birth, diary month and cohort. Model 2 was performed in the same way as model 1 along with 14 bacterial and fungal genera that were simultaneously adjusted. In the analyses of healthy weeks or antibiotic use, the total number of diary weeks was 15 087 and in the analyses of otitis or fever weeks 14 678 in 354 children.

Supplemental Table E4. Adjusted associations between relative abundances of dog-associated 12 bacterial or 2 fungal genera as well as sum of selected genera in dust samples and healthy weeks, usage of antibiotics, weeks of otitis or fever in children aged 2-12 months of age.

aOR adjusted Odds Ratios; 95% CI, Confidence intervals were obtained by using GEE analysis, working correlation matrix AR(1); p-values for microbial genera/sum variables are from linear trend test; p-values <0.05 are in bold. DL detection limit; Genera /sum variables were divided into tertiles/ categorized into three classes (under detection level, and two equal classes) and the lowest category is the reference category; Models are adjusted for gender, birth weight, number of siblings, living environment, season of birth, diary month, maternal smoking and parental atopy and cohort. Total number of diary weeks in bacterial analyses in healthy weeks and usage of antibiotics is 15 644 and in otitis and fever weeks is 15 226 and in fungal analyses the similar values are 15 129 and 14 720. The total number of children with diary data were 367 in bacterial and 355 in fungal analyses. The sum of relative abundances of selected genera were Clostridium, Collinsella, Eubacterium candidate group, Helicobacter, Megamonas, Pasteurella, and Ruminococcus candidate group in healthy weeks; Collinsella and Pasteurella in usage of antibiotics; and Collinsella, Pasteurella, Ruminococcus candidate group and Udeniomyces in weeks of fever. ^a^No sum variable for otitis was created.

Supplemental Table E5. Adjusted associations between dog keeping and healthy weeks, usage of antibiotics, or fever weeks, and mutually adjusted for sum of selected bacterial and fungal genera in dust from living room floor.

aOR Adjusted Odds Ratios; 95% CI Confidence intervals obtained by using GEE analysis, working correlation matrix AR(1); p p-values for sum variables are from linear Trend test; p-values <0.05 are in bold. Models are adjusted for gender, birth weight, living environment (farm, rural non-farm, suburban), number of siblings, maternal smoking, parental atopy, breastfeeding (solely, partly, no), season of birth, diary month and cohort. % how many percent given sum variable explains the effect between dog keeping and health weeks, usage of antibiotics or fever weeks; The sum of relative abundances of selected bacterial genera were *Clostridium, Collinsella, Eubacterium* candidate group, *Helicobacter, Megamonas, Pasteurella*, and *Ruminococcus* candidate group in healthy weeks*; Collinsella* and *Pasteurella* in usage of antibiotics; and *Collinsella, Pasteurella, Ruminococcus* candidate group and *Udeniomyces* in weeks of fever. No sum variable for otitis was created.

Supplemental Table E6. Adjusted associations between dog keeping and healthy weeks, usage of antibiotics, weeks of otitis or fever, additionally adjusted for bacterial signatures in house dust.

aOR Adjusted Odds Ratios; 95% CI Confidence intervals obtained by using GEE analysis, working correlation matrix AR(1); p* p-values are from linear Trend test; p-values <0.05 are in bold. Models are adjusted for gender, birth weight, living environment (farm, rural non-farm, suburban), number of siblings, maternal smoking, parental atopy, breastfeeding (solely, partly, no), season of birth, diary month and cohort. The signatures were divided into tertiles and the lowest tertile is the reference category, except human sourced bacteria, which were continues and are expressed as change per interquartile range (IQR) change. expl.(%) percentage how much the given bacterial signature explains the association between dog keeping and the given outcome. If expl.(%) is missing, the signature did not explain the association. Total number of diary weeks in healthy weeks and usage of antibiotics is 15 644 and in otitis and fever weeks 15226, which correspond to the numbers of 367 children.

Supplemental Table E7. Adjusted associations between dog keeping and healthy weeks, usage of antibiotics, weeks of otitis or fever, additionally adjusted for fungal richness in house dust.

aOR Adjusted Odds Ratios; 95% CI Confidence intervals obtained by using GEE analysis, working correlation matrix AR(1); p* p-values for fungal richness are from linear Trend test; p-values <0.05 are in bold. Models are adjusted for gender, birth weight, living environment (farm, rural non-farm, suburban), number of siblings, maternal smoking, parental atopy, breastfeeding (solely, partly, no), season of birth, diary month and cohort. Fungal richness was divided into tertiles and the lowest tertile is the reference category. Total number of diary weeks in healthy weeks and usage of antibiotics is 15 129 and in otitis and fever weeks 14 720, which correspond to the numbers of 355 children. Fungal richness did not explain the association between dog keeping and four outcomes.

Supplemental Table E8. Additional characteristics and comparisons between households with and without dogs.

N the number of observations, n the number of observations in the given group, % the percentage of the observations within dog households and without dog. P-values are from chi square test.* Only asked in LUKAS2 cohort.

Supplemental Table E9. Adjusted associations between dog keeping and healthy weeks, usage of antibiotics, weeks of otitis or fever, additionally adjusted for bacterial and fungal genera in dust from living room floor (separate class for missing added).

aOR adjusted Odds Ratios, 95% CI, Confidence interval, p values are obtained by using GEE analysis, working correlation matrix AR(1).Models are adjusted for sex, living environment (farm, rural non-farm, suburban), number of siblings, maternal smoking, parental atopy, breastfeeding (solely, partly, no), birth weight, season of birth, diary month and cohort. Expl.% how many percent given genus explain the effect between dog contact and health weeks, usage of antibiotics, or otitis or fever weeks; Total number of diary weeks in bacterial analyses in healthy weeks and usage of antibiotics is 15 644 and in otitis and fever weeks is 15 226 and in fungal analyses the similar values are 15 129 and 14 720, which correspond to the numbers of 397 children.

Supplemental Table E10. Adjusted associations between dog keeping and healthy weeks, usage of antibiotics, weeks of otitis or fever, additionally adjusted for bacterial and fungal genera in dust from living room floor (missing input at random).

aOR adjusted Odds Ratios, 95% CI, Confidence interval, p values are obtained by using GEE analysis, working correlation matrix AR(1).Models are adjusted for sex, living environment (farm, rural non-farm, suburban), number of siblings, maternal smoking, parental atopy, breastfeeding (solely, partly, no), birth weight, season of birth, diary month and cohort. Expl.% how many percent given genus explain the effect between dog contact and health weeks, usage of antibiotics, or otitis or fever weeks; Total number of diary weeks in bacterial analyses in healthy weeks and usage of antibiotics is 15 644 and in otitis and fever weeks is 15 226 and in fungal analyses the similar values are 15 129 and 14 720, which correspond to the numbers of 397 children.


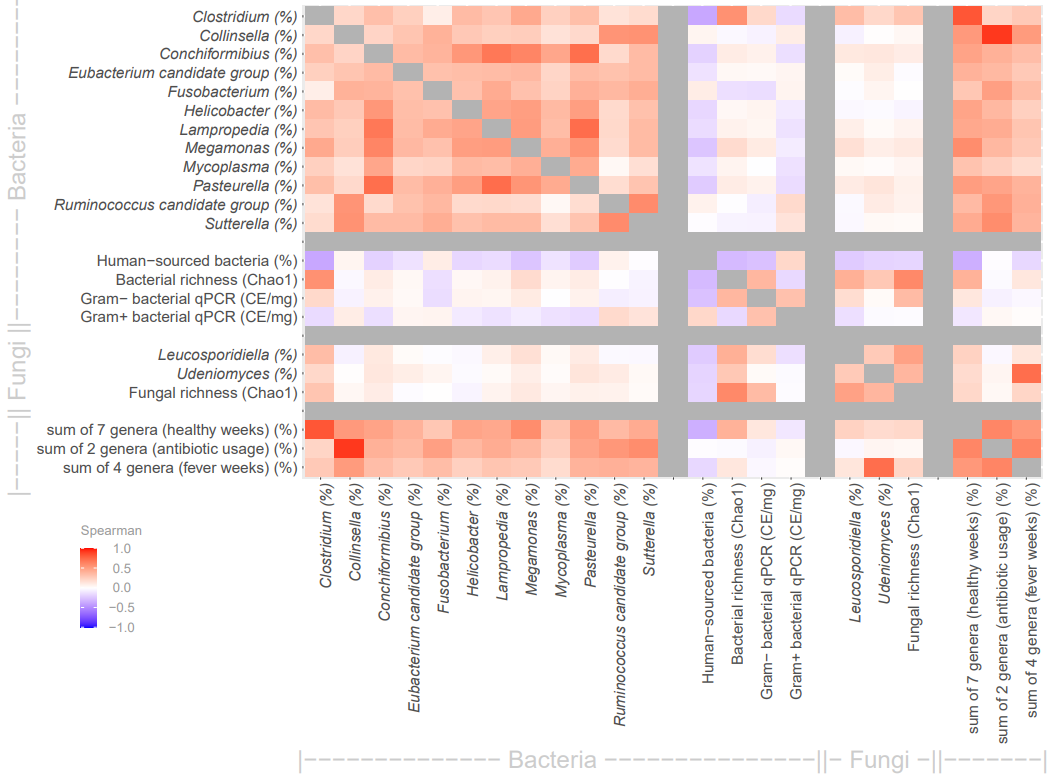


Supplemental Figure E1. Heat map of Spearman correlations between bacterial and fungal signatures or genera in LUKAS cohorts (LUKAS1+2) of 394 dust samples. Heat map was created using R software. (%) defines relative abundance; qPCR quantitative polymerase chain reaction; CE cell equivalent; mg per milligram of dust. The sum of relative abundances of genera was *Clostridium, Collinsella, Eubacterium* candidate group, *Helicobacter, Megamonas, Pasteurella*, and *Rumninococcus* candidate group in healthy weeks*; Collinsella* and *Pasteurella* in usage of antibiotics; and *Collinsella, Pasteurella, Rumninococcus* candidate group and *Udeniomyces* in weeks of fever. No sum variable for otits was created.


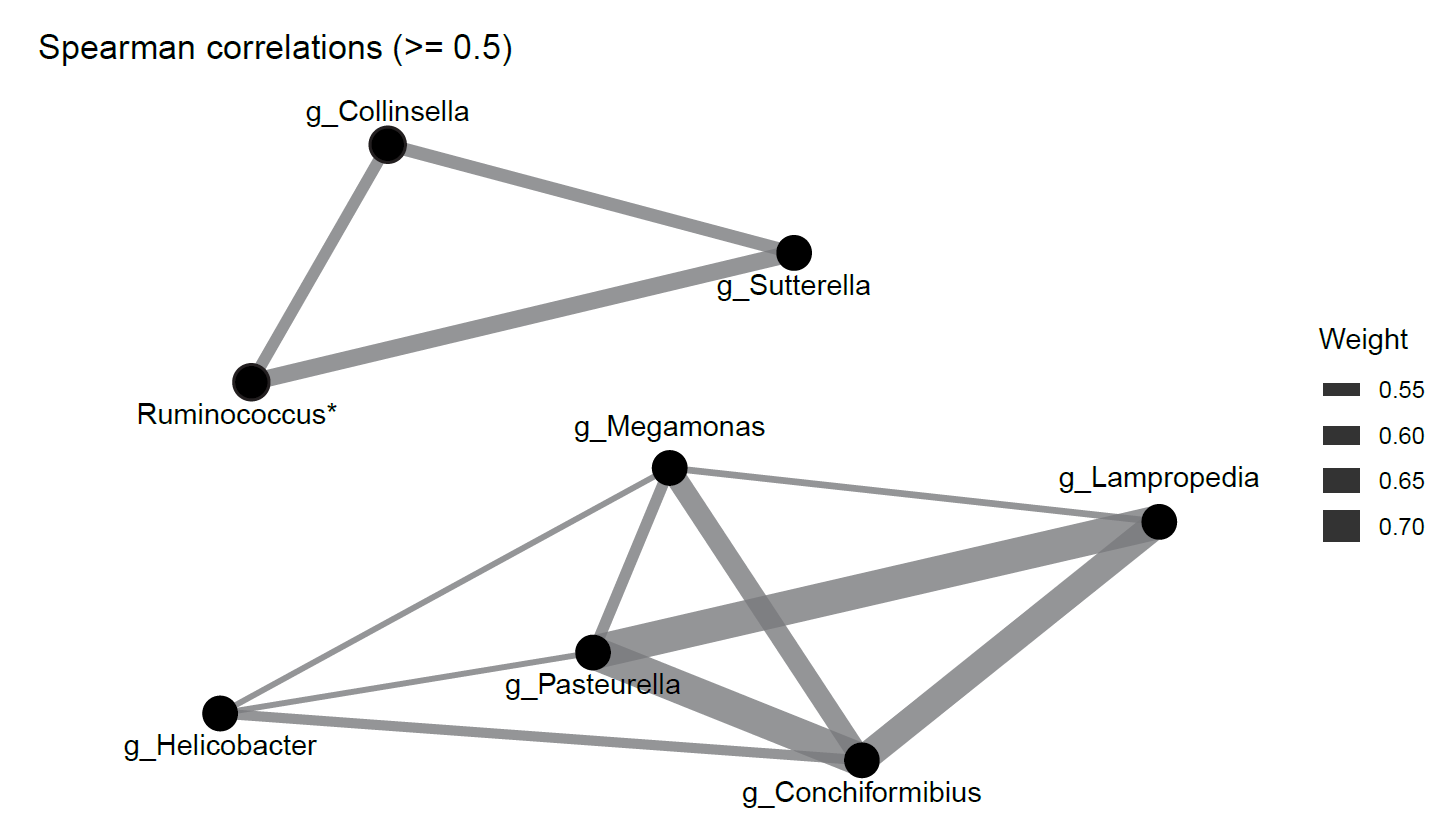


Supplemental Figure E2. Result from the correlation-based network analyses. Group A consist of *Collinsella, Ruminococcus* candidate group (*) and *Sutterella* and the group B *Lampropedia, Pasteurella* and *Conchiformibius*.
